# Birmingham General Dispensary

**Published:** 1830-06

**Authors:** 


### BIRMINGHAM GENERAL DISPENSARY.

### *Case of Retroverted Uterus, treated by Puncture of that Organ.* By J. M. Baynham, Surgeon to the General Dispensary and to the Town Infirmary of Birmingham.*

The consequences of retroversion of the uterus have been so often fatal, that a case successfully treated by surgical operation cannot be devoid of interest. The practice adopted in this instance will be found uncommon; and, since it led to a successful issue under the most unpromising circumstances, deserves to be recorded.

Hannah Martin, aged thirty, of spare make, was admitted a patient of the dispensary, 28th of March, 1828. She was then in the sixth month of her second pregnancy, the history of her case to which period is briefly as follows:

When employed six weeks previously in moving a heavy weight, she suddenly felt acute pain in the lower part of the belly. To this, however, little importance was attached at the moment. Two days afterwards retention of urine occurred, with almost constant pain. The nature of the case appears to have been overlooked by the gentleman consulted in the first instance, since the use of a catheter was not proposed. She had dripping of urine, with progressive enlargement of the abdomen during the next month; at the end of which time, finding no relief in medicine, she applied to another surgeon, who, by the introduction of a catheter, obtained eight pints of urine in the morning, and nearly the same quantity seven hours afterwards. No examination, per vaginam, was even now instituted, and, of course, no permanent relief secured to the patient, the catheter only being used night and morning in the next fortnight. When recommended to the dispensary, she had kept her bed three weeks, and was in a state of high fever; her pulse 136, short and indistinct. She had frequent vomiting, constant micturition, tenesmus, fulness, tension, and tenderness of the abdomen.

In my first attempts to pass a catheter, I was embarrassed by the altered state of the external organs; a large portion of the vagina being prolapsed, and the clitoris and nymphæ greatly enlarged. The urine which escaped by the instrument resembled

* Edinburgh Med. and Surg. Journal, April 1830.

the contents of a psoas abscess, but was much more fetid. The entire cavity of the pelvis was occupied by a tumor, which caused protrusion of the anus, and also eversion of the lower extremity of the bowel. The mouth of the uterus was far beyond the reach of the finger, and the fundus of this organ was situated less than one inch from the anus; a circumstance which rendered the admission of the finger into the rectum a work of much difficulty. Feeling satisfied that no urine remained in the bladder, I attempted to replace the uterus by a gradual introduction of the whole hand into the vagina. The os uteri pointed directly upwards, and was raised above the pubis: in fact, the retroversion was complete.

Having persevered as long as seemed consistent with the safety of the patient, I requested the attendance of two of my colleagues, and they met me in consultation the same afternoon, (March 28.) She had become much more exhausted and restless. Her anxiety of manner, and the failure of her pulse, leading us to suppose that she was nearly moribund, I proposed the immediate introduction of a trocar into the uterus, for the purpose of lessening its volume. Preparatory to any other steps, the catheter was again used, and having then placed the woman upon her elbows and knees, I once more endeavoured to raise the tumor, but not succeeding better than before, I slowly passed my hand into the rectum, and, adapting it as far as possible to the base of the tumor, continued for some time to make the firmest pressure, without sensible advantage.

Mr. Blount, one of the gentlemen present, in the expectation of a better result, desired to satisfy himself of the impracticability of success before the operation of puncture was adopted. Having passed his finger into the os uteri, he endeavoured to rupture the membranes; but, although assisted by a curved metallic instrument, he was compelled to relinquish his purpose, and all other expedients to relieve the patient having failed, it was determined to employ the trocar. In this proceeding I selected the most prominent point of the tumor in the rectum.* The entrance of the trocar not being followed by any discharge, it was withdrawn, and introduced a second time in nearly the same situation. About twelve ounces of colourless fluid now escaped by the canula, but not without frequently changing its position; since the opening was at times obstructed by the presence of the child. The fulness of the uterus having thus been diminished, attempts were again made to

* Mr. Baynham states, in a subsequent part of his communication, that although in this case the operation was performed by the rectum, he does not consider this an eligible situation. It was selected because the uterine tumor may be said to have pointed most distinctly in the bowel. Perforation of the uterus through the vagina he deems preferable, since, without an equivalent advantage, even so small a wound of the intestine ought to be avoided. Moreover, Mr. B. remarks, there will be less probability of injuring the placenta, which is usually attached to the fundus, and trifling as the chance may be of preserving the fœtus, it is entitled to consideration.

carry it above the brim of the pelvis, and this was affected in less than a quarter of an hour. When the organ had recovered its proper situation, the os uteri was found partially dilated, and the membranes somewhat protruding. A full opiate was prescribed, and the woman passed a better night than any in the previous month.

The next morning, although still in a state of great exhaustion, she was decidedly improved. Labour pains occurred in the evening of the 29th, and less than one hour sufficed, fortunately without hemorrhage, to exclude the contents of the uterus, twenty-five hours after the operation. The ovum was entire, the membranes perfect, and still retaining ten ounces of liquor amnii untinged with blood. The fœtus was perfectly fresh, and of the ordinary size at six months. The trocar both times had penetrated the substance of the placenta near to the insertion of the cord, and once had entered the abdomen of the child; forming an aperture through which nearly the whole of the small intestines were forcibly protruded by the pressure subsequently used. The second puncture was referrible to this unavoidable accident. It is worthy of remark, that, notwithstanding the placenta was twice perforated, hardly a teaspoonful of blood was lost.

The catheter was used but once after this time, when a pint of equally offensive urine was evacuated. Incontinence then supervened, and lasted nearly five weeks; and severe pains continued to be felt in the pelvis for some time. Copious vaginal discharge, added to the stillicidium urinæ, kept up a state of soreness and excoriation; and it was not until after a month that her urine lost its fetor. Considerable masses of coagulated lymph were often discharged, and at separate times four pieces of regularly organized membrane, which were mistaken for portions of the bladder, but which subsequent events happily proved to be parts of the vagina only. At the end of April she had the satisfaction of holding small quantities of urine, and in a fortnight could retain it almost as well as before her illness. The rectum was longer in recovering its tone than the vagina; purulent evacuations taking place from the former passage, with frequent and sometimes distressing tenesmus, until after she was in other respects well. It is probable that an abscess formed in the cellular substance, between the vagina and rectum, since the matter voided per anum was different, and more in quantity than the mere surface of the bowel could have yielded. She kept her bed three weeks before she applied to the dispensary, and did not leave it until nearly a month afterwards. Upon the 7th of May, she was sufficiently recovered to leave home and engage in her usual occupation. Menstruation occurred in the first week of June, and she has continued in good health since that period.*

* Mr. Baynham adds to the relation of this interesting case many important remarks, for which we must refer to the original.—EDITOR.

